# Mapping the neuroanatomical impact of very preterm birth across childhood

**DOI:** 10.1002/hbm.24847

**Published:** 2019-11-05

**Authors:** Marlee M. Vandewouw, Julia M. Young, Sarah I. Mossad, Julie Sato, Hilary A. E. Whyte, Manohar M. Shroff, Margot J. Taylor

**Affiliations:** ^1^ Department of Diagnostic Imaging Hospital for Sick Children Toronto Ontario Canada; ^2^ Program in Neurosciences & Mental Health Hospital for Sick Children Toronto Ontario Canada; ^3^ Department of Psychology University of Toronto Toronto Ontario Canada; ^4^ Division of Neonatology Hospital for Sick Children Toronto Ontario Canada; ^5^ Department of Paediatrics University of Toronto Toronto Ontario Canada; ^6^ Department of Medical Imaging University of Toronto Toronto Canada

**Keywords:** brain structure, child development, magnetic resonance imaging, preterm birth

## Abstract

Those born very preterm (VPT; <32 weeks gestational age) have an increased risk in developing a wide range of cognitive deficits. In early‐to‐late childhood, brain structure has been shown to be altered in VPT compared to full‐term (FT) children; however, the results are inconsistent. The current study examined subcortical volumes, cortical thickness, and surface area in a large cohort of VPT and FT children aged 4–12 years. Structural magnetic resonance imaging (MRI) was obtained on 120 VPT and 146 FT children who returned up to three times, resulting in 176 VPT and 173 FT unique data points. For each participant, Corticometric Iterative Vertex‐based Estimation of Thickness was used to obtain global measurements of total brain, cortical grey and cortical white matter volumes, along with surface‐based measurements of cortical thickness and surface area, and Multiple Automatically Generated Templates (MAGeT) brain segmentation tool was used to segment the subcortical structures. To examine group differences and group–age interactions, mixed‐effects models were used (controlling for whole‐brain volume). We found few differences between the two groups in subcortical volumes. The VPT children showed increased cortical thickness in frontal, occipital and fusiform gyri and inferior pre–post–central areas, while thinning occurred in the midcingulate. Cortical thickness in occipital regions showed more rapid decreases with age in the VPT compared to the FT children. VPT children also showed both regional increases, particularly in the temporal lobe, and decreases in surface area. Our results indicate a delayed maturational trajectory in those born VPT.

## 1 INTRODUCTION

Very preterm (VPT) birth, defined as birth before 32 weeks gestational age (GA), has been associated with widespread impairments across cognitive domains in childhood that persist into adulthood (Anderson, 2014). Understanding the underlying neural bases of these difficulties across development is crucial for understanding the critical periods for effective interventions and identifying those at higher risk for developing later cognitive/behavioural problems. In the third trimester, brain growth is occurring very rapidly with marked increases in cortical surface area relative to volume as cortical folding becomes more complex. Preterm birth occurs during this critical period and may disturb neurodevelopment. Such perturbation could have lasting impacts on brain structure, including volumes of white and gray matter, surface area and cortical thickness.

Over childhood the brain continues to grow, with gray matter increasing, seen as increasing cortical thickness and surface area, over the early years, and then decreasing cortical thickness after mid‐childhood (e.g., Mills et al., 2016; Raznahan et al., 2011), as a result of cortical pruning as well as increased myelination (Paus, 2005). White matter increases steadily into the third decade of life. Subcortical structures show variable maturational changes through childhood and adolescence (Sussman, Leung, Chakravarty, Lerch, & Taylor, 2016; Wierenga et al., 2014). These developmental patterns can be used to predict brain maturation and relate to behaviour (Khundrakpam et al., 2015; Lewis et al., 2018). There has thus been considerable interest in understanding these developmental trajectories in VPT born children, as an indicator or predictor of the cognitive difficulties that many experience. Although a number of investigators have pursued analyses of various structural brain metrics, the results, summarized below, do not show consistent patterns.

Progressive and regressive changes occur in cortical and deep gray matter during the period of development spanning earlier (3–8 years of age) and later (8–12 years of age) childhood (e.g., Coupé, Catheline, Lanuza, & Manjón, 2017; Sussman, Leung, Chakravarty, Lerch, & Taylor, 2016; Wierenga et al., 2014). From late infancy into earlier childhood, VPT children have been shown to have comparable brain development with their full term (FT) peers with regards to total brain volume (TBV), cortical surface area and cortical thickness (Phillips et al., 2011). However, differences begin to emerge in earlier childhood, with preterm children showing volumetric decreases in the cortical gray matter, white matter and subcortical gray matter volume compared to their FT counterparts (Monson et al., 2016; Sølsnes et al., 2016), suggesting delayed maturation during this time period. These volumetric decreases have been found to extend into late childhood (Grunewaldt et al., 2014; Kesler et al., 2008; Lax et al., 2013; Soria‐Pastor et al., 2009), yet increases in the percentage of gray matter volume in parietal, frontal and occipital horn regions in VPT compared to FT children have also been reported (Kesler et al., 2004). Over late childhood, the typical volumetric changes across age in grey and white matter have been reported to be reduced in VPT children (Ment et al., 2009), and significant relations between subcortical volumes and age existed in the FT children, but not the VPT children (Lax et al., 2013), consistent with widespread, less mature brain structures.

Total surface area and cortical thickness have been reported to be reduced in VPT children (Lax et al., 2013), while local decreases in surface area have been reported in the bilateral temporal and left pre‐ and post‐central cortices in VPT compared to FT children (Zhang et al., 2015), yet other brain areas, including the right parietal and frontal regions, the bilateral cingulate cortices and precuneus showed increased surface area (Zhang et al., 2015). Similarly, studies have found both regional cortical thickness decreases (Lax et al., 2013; Sølsnes et al., 2015; Zubiaurre‐Elorza et al., 2012) and increases (Sølsnes et al., 2015) in VPT compared to FT children. There is some evidence to suggest delayed maturation in cortical thinning in VPT children in the earlier years with some catch‐up in later childhood, with local increases in cortical thickness in VPT children only emerging when children less than 10 years old were considered (Mürner‐Lavanchy et al., 2014). Furthermore, across early to late childhood, VPT children showed negative relations between age and cortical thickness in the right frontal, parietal and inferior temporal regions only in the preterm children, while no correlations with age were found in the FT children (Mürner‐Lavanchy et al., 2014).

Thus, although there are many studies showing structural brain differences between VPT and FT children, the results are not consistent. One reason may be that many studies had a narrow age range (sometime only over 1 year) and the brain changes over childhood are complex and often not linear, while others had small samples. Also, often differing metrics were used to assess neuroanatomical differences. To clarify these discrepant findings in the literature, we investigated the brain structure in a large cohort of 200 VPT children spanning four to 12 years of age, and compared their developmental trajectories to matched FT‐born children. Whole brain, gray and white matter volumes, subcortical volumes, cortical thickness, and surface area were all compared between the groups to fully characterize patterns of brain development from early to later childhood. Given the literature, we hypothesized that with age, the VPT group would show developmental rates that were similar to the full term controls, but would not show catch‐up, having generally smaller volumes (cortical gray, white, and deep gray matter) and/or reduced cortical thickness and surface areas across the age range.

## 2 METHODS

### 2.1 Participants

The data included in the analysis (passing quality control, see the image processing section for further details) consisted of 383 scans: 201 magnetic resonance imaging (MRI) scans from VPT and 182 from FT children. As part of various studies at the Hospital for Sick Children in Toronto, 137 VPT children and 154 full term (FT; born at >37 GA) underwent up to three longitudinal structural MRI scans between 4–12 years of age. A total of 182 structural MRIs were obtained from the FT children (128 participants with one time point, 24 participants with two time points, and two participants with three time points), and 201 from the VPT children (98 participants with one time point, 14 participants with two time points, and 25 with three time points). For the FT children, the absence of prematurity, and learning, language, neurological and developmental disabilities were a requirement for initial recruitment, while VPT children with known chromosomal or major congenital abnormalities were excluded. At each time point, children underwent neuropsychological assessments. IQ was measured using the Wechsler Preschool and Primary Scales of Intelligence—Third Edition (WPPSI‐III; Wechsler, 2002) for children ≤5 years and the Wechsler Abbreviated Scale of Intelligence for children ≥6 years (WASI; Wechsler, 1999). Parents gave written consent and the children gave verbal assent. All study protocols were approved by the research ethics board at the Hospital for Sick Children. In the VPT cohort, clinical radiological review at birth reported that 37% had some brain injury (defined as the presence of at least one of the following findings at birth: echodense intraparenchymal lesions, white matter lesions, periventricular leukomalacia, porencephalic cysts, and ventriculomegaly with or without intraventricular hemorrhage).

### 2.2 Image acquisition

T1‐weighted imaging data were collected using three‐dimensional magnetization prepared rapid gradient echo (MPRAGE) protocols on either a 3T Siemens MAGNETOM Trio scanner with a 12 channel head coil (TR/TE/TI: 2300/2.96/900 ms; FA: 9°; FOV: 240 × 256 mm; number of slices: 192; resolution: 1.0 mm isotropic; scan time: 5:03 min) or a 3T Siemens MAGNETOM PrismaFIT with a 20 channel head and neck coil (TR/TE/TI: 1870/3.14/945 ms; FA: 9°; FOV: 240 × 256 mm; number of slices: 192; resolution: 0.8 mm isotropic; scan time: 5:01 min).

### 2.3 Image processing

T1‐weighted images were filtered using a spatial adaptive nonlocal means denoising filter (Manjón, Coupé, Martí‐Bonmatí, Collins, & Robles, 2010) and processed through both cortical and subcortical pipelines.

For the cortical pipeline, Corticometric Iterative Vertex‐based Estimation of Thickness (CIVET, version 2.1.0; Lerch & Evans, 2005) was used on the CBRAIN platform (Sherif et al., 2014). The T1‐weighted images were corrected for nonuniformities (Sled, Zijdenbos, & Evans, 1998) and registered to the ICBM152 nonlinear sixth generation template using stereotaxic registration (Collins, Neelin, Peters, & Evans, 1994; Grabner et al., 2006). Brain tissue was masked (Smith, 2002) and classified into gray matter, white matter and CSF (Tohka, Zijdenbos, & Evans, 2004; Zijdenbos, Forghani, & Evans, 1998). High‐resolution surfaces of each hemisphere were extracted using the Constrained Laplacian Anatomic Segmentation using Proximity (CLASP; Kim et al., 2005; MacDonald, Kabani, Avis, & Evans, 2000) method. Cortical thickness was measured in native space as the distance between the gray and white matter surface boundaries (Lerch & Evans, 2005) and smoothed using a 30 mm diffusion kernel (Boucher, Whitesides, & Evans, 2009). The surfaces were registered to the MNI ICBM152 surface template and the transformations were used to interpolate the cortical thickness data onto the surface template (Boucher, Whitesides, & Evans, 2009; Lyttelton, Boucher, Robbins, & Evans, 2007; Robbins, 2004). Vertex‐based surface area, which measures local variations of area relative to the vertex distribution on the surface template, was computed on the resampled surfaces. TBV, gray matter volume and white matter volume were also extracted for each subject. Gray and white matter volumes were converted to a percentage of the TBV, as there is substantial literature showing that VPT‐born individuals have smaller TBV. Quality control was performed by visually inspecting each participant's brain mask, registration to the template, tissue classification and brain segmentation (following this, 34 subjects were excluded).

For the subcortical pipeline, Multiple Automatically Generated Templates (MAGeT) brain segmentation tool (Chakravarty et al., 2013; Pipitone et al., 2014) was used to segment each subject's subcortical structures. Prior to processing, bias field correction was performed on the T1‐weighted images using the N4 algorithm (Tustison et al., 2010), and the resulting images were cropped for excess non‐head features. Five manual segmentations of the amygdalae (Treadway et al., 2015), hippocampi (Winterburn et al., 2013), striati, globus pallida, and thalami (Tullo et al., 2018) were nonlinearly warped to a 21 image subset of the participants' T1‐weighted images, equally distributed across groups, called the template library. The resulting five segmentations for each template image were propagated to the remaining images in the sample, and fused via majority voting, which assigns each voxel its most frequently occurring segmentation label. Visual inspection of each participant's subcortical segmentations was performed to ensure accuracy between the original structural image and the resulting labels. For each subject, the volumes of the five structures were extracted, summed across hemisphere, and converted to a percentage of TBV (calculated from the cortical pipeline).

### 2.4 Statistics

To test differences between the VPT and FT datasets by age, a Mann–Whitney *U* test was used due to the non‐normality of the age distribution. Chi‐squared tests were used to test for differences in sex ratio and scanner ratio (ratio of scans acquired on the Trio to PrismaFIT).

To utilize the longitudinal data while maximizing statistical power, mixed‐effects models were used to investigate group differences and interactions between the VPT and FT children in whole‐brain volumes, subcortical volumes, cortical thickness and surface area across age. In each model, each participant was modeled as a random effect, and sex and scanner were used as nuisance covariates in all models.

First, a mixed‐effects model was used to test the relation between age and TBV and percentage volumes of the gray and white matter (Model 1):

$$Y=\text{Group}+\mathrm{Age}+\text{Group}\times \mathrm{Age}+\mathrm{Sex}+\text{Scanner}+\mathrm{rand}\left(\text{Subject}\right)+\text{Intercept}$$

with significance held at *p* < .05. The effect of age on the percentage volume of each of the five subcortical structures was then investigated using Model 1. The resulting *p* values were false discovery rate (FDR) corrected (Benjamini & Hochberg, 1995) across each term (group, age, group‐by‐age, sex, and scanner) in the five mixed‐effects models to account for multiple‐comparisons across the subcortical structures, and significance was held at *q* < .05. Finally, within the VPT group, the effect of GA on the normalized subcortical volumes was investigated while controlling for age (Model 2):

$$Y=\mathrm{GA}+\mathrm{Age}+\mathrm{Sex}+\text{Scanner}+\mathrm{rand}\left(\text{Subject}\right)+\text{Intercept}$$

and FDR‐correcting across terms. All analyses were performed in MATLAB (The Mathworks Inc., 2016).

Next, group differences and interactions in the relations between age and cortical thickness and surface area were analyzed using the MATLAB (The Mathworks Inc., 2016) toolbox SurfStat (http://www.math.mcgill.ca/keith/surfstat/). Mixed effects models (Model 3):

$$Y=\text{Group}+\mathrm{Age}+\text{Group}\times \mathrm{Age}+\mathrm{TBV}+\mathrm{Sex}+\text{Scanner}+\mathrm{rand}\left(\text{Subject}\right)+\text{Intercept}$$

were used to test the relations between age and both cortical thickness and surface area at each vertex, with TBV as a nuisance covariate. Clusters were corrected for multiple comparisons using whole‐brain random field theory (RFT) and thresholded at *p* < .05. The relations between GA and cortical thickness and surface area were also investigated while controlling for age and correcting using RFT (Model 4):

$$Y=\mathrm{GA}+\mathrm{Age}+\mathrm{TBV}+\mathrm{Sex}+\text{Scanner}+\mathrm{rand}\left(\text{Subject}\right)+\text{Intercept}$$

The vertices of significant clusters were labeled using the automated anatomical labeling (AAL) atlas (Tzourio‐Mazoyer et al., 2002), and AAL regions with more than 100 overlapping vertices were reported.

## 3 RESULTS

### 3.1 Participant demographics

After quality control, 176 T1‐weighted images of VPT children (120 unique VPT participants: 86 with one time point, 12 with two time points, and 22 with three time points) and 173 T1‐weighted images of FT children (146 unique FT participants: 121 with one time point, 23 with two time points, and two with three time points) remained. There were no significant differences in age (*U* = 11,600; *p* = .06), sex (χ^2^ = 0.34, *p* = .56) or scanner (χ^2^ = 01.43, *p* = .23) between the two groups. The FT children had significantly higher IQ than those born VPT (*U* = 8,299; *p* = 4.87e^−12^). The demographics of the two groups are summarized in Table 1.

**Table 1:** Participant demographics, where *N*_Total_ is the total number of data sets, and *N*_1_, *N*_2_, and *N*_3_ are the number of participants with one, two, and three time points, respectively

	*N*_Total_ (*N*_1_:*N*_2_:*N*_3_)	Age (mean ± *SD*)	Sex (M:F)	Scanner (Trio:PrismaFIT)	IQ (mean ± *SD*)	GA (weeks; mean ± *SD*)
VPT	176 (86:12:22)	6.76 ± 2.18	95:81	103:73	101.51^a^ ± 16.08	28.26 ± 1.73
FT	173 (121:23:2)	7.17 ± 2.23	88:85	112:61	112.63^b^ ± 24.12	>37

Abbreviations: F, female; FT, full‐term; GA, gestational age; IQ, intelligence quotient; M, male; VPT, very preterm.

a Data missing for one VPT data set.

b Data missing for six FT data sets.

**Figure 1:**
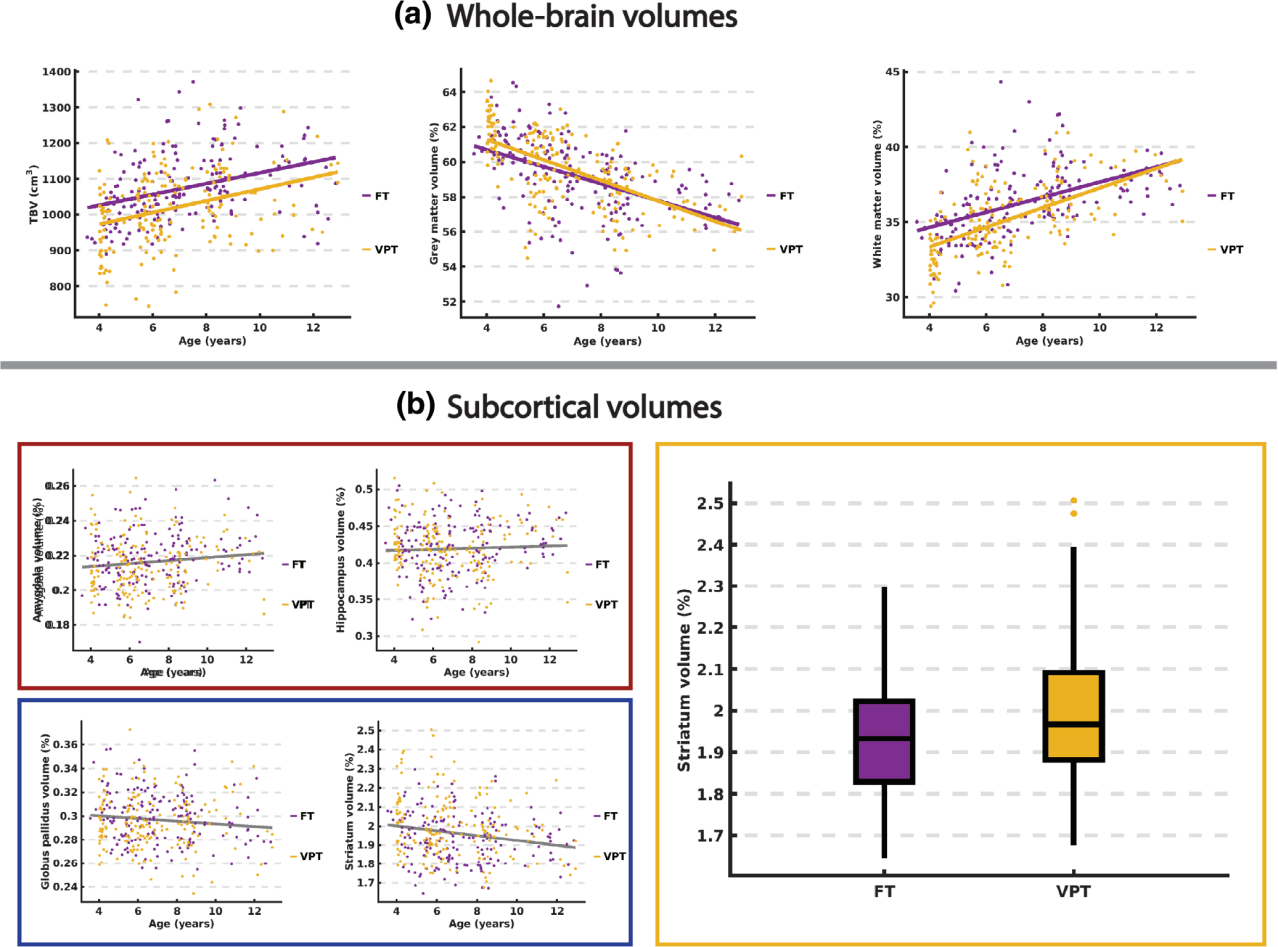
(a) Significant (*p* < .05) effects of group, age, and group‐by‐age interactions on TBV (i), percent of gray matter volume (ii), and percent of white matter volume (iii). (b) Significant effects of age (i, ii, *p* < .05 uncorrected) and group (iii, *q* < .05 corrected) on cortical thickness in the subcortical structures

There was a significant effect of both group (*t* = 3.33, *p* = 9.69e^−4^) and age (*t* = 5.28, *p* = 2.25e^−7^) on TBV, with TBV being higher in the FT children and increasing with age in both groups (Figure 1ai). Across both the VPT and FT children, the percentage volume of gray matter was found to decrease significantly with age (*t* = 8.89, *p* = 2.78e^−17^; Figure 1aii), while the percentage volume of white matter was found to increase significantly with age (*t* = 8.91, *p* = 3.11^−17^; Figure 1aiii). Furthermore, VPT children were found to have lower percentage of white matter compared to FT children across age (*t* = 3.38, *p* = 8.22e^−4^), and there was a marginally significant group‐by‐age interaction (*t* = 1.98, *p* = .05), with percentage of white matter volume increasing more with age in VPT children. Neither TBV nor percentage of gray or white matter volume correlated with GA.

#### 3.1.1 Subcortical volumes

Across groups, there were no significant (*q* < .05, corrected) effects of age on the percentage volume of the subcortical structures; however, uncorrected, the percentage volume of the amygdalae (*t* = 2.77, *p* = 5.99e^−3^) and hippocampi (*t* = 2.14, *p* = .03) were found to increase with age (Figure 1bi), while the percentage volume of the striati (*t* = 2.08, *p* = .04) and globus pallida (*t* = 2.03, *p* = .04) were found to decrease with age (Figure 1bii). In VPT children, the percentage volume of the striati was found to be significantly larger compared to FT children (*t* = 3.31, *q* = .01; 1.99 ± 0.16% [VPT] 1.93 ± 0.13% [FT]). There were no significant group‐by‐age interactions, nor a significant effect of GA in any of the subcortical structures after FDR‐correction.

#### 3.1.2 Cortical thickness

**Figure 2:**
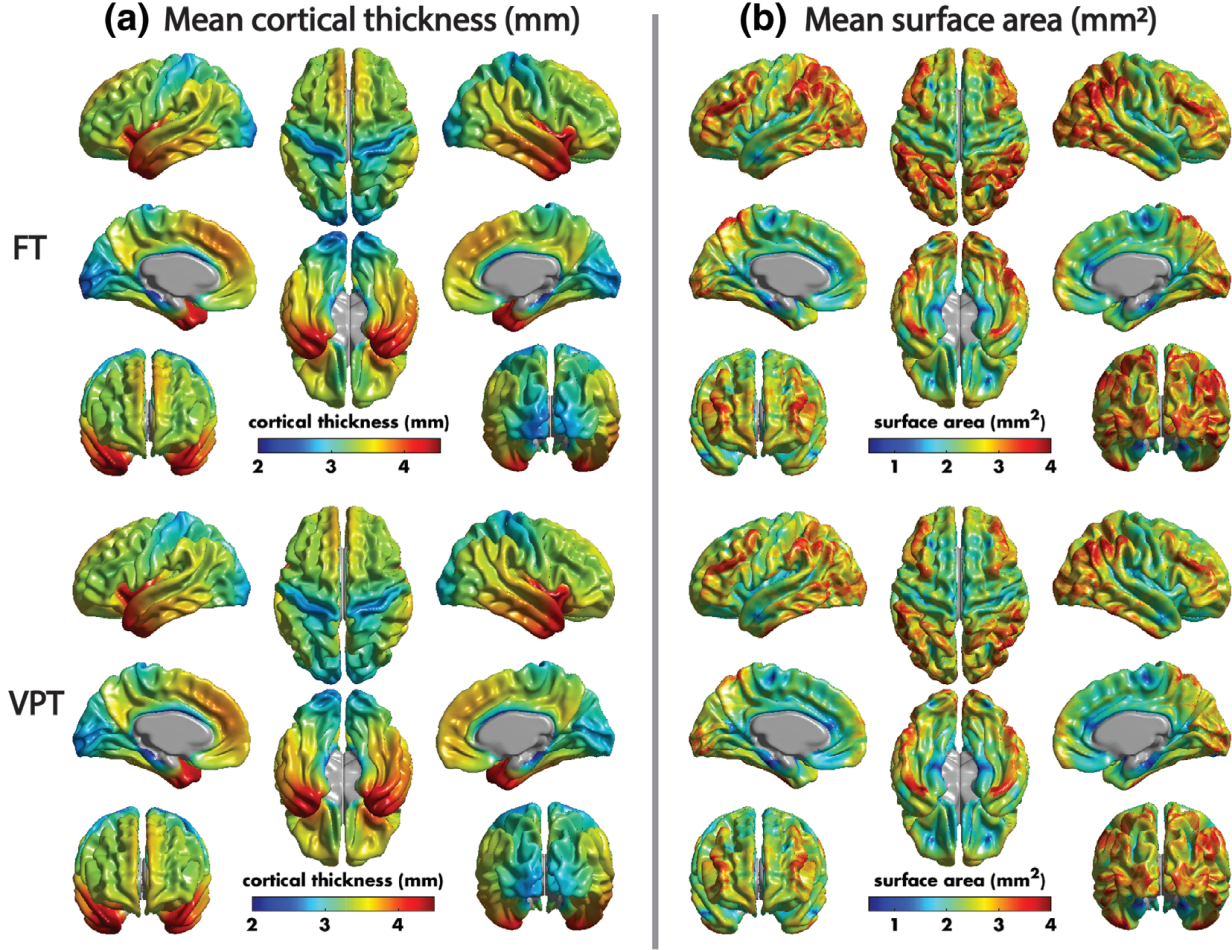
Mean cortical thickness (a) and surface area (b) across the VPT and FT children. FT, full‐term; VPT, very preterm

**Figure 3:**
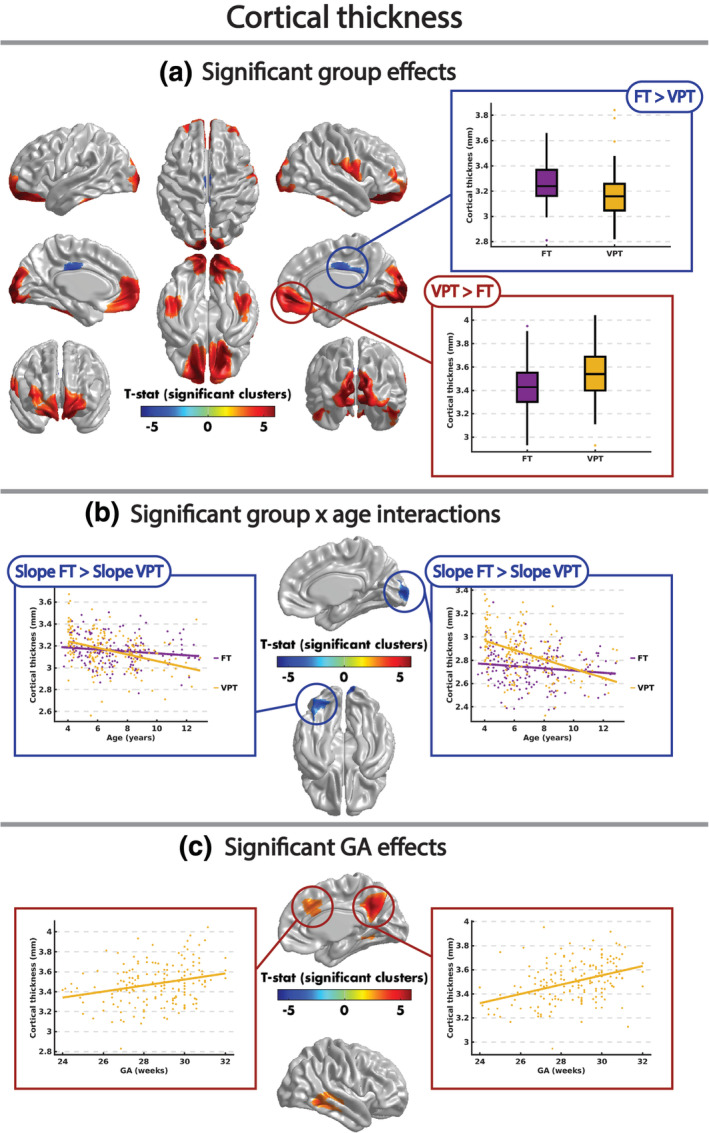
Significant (RFT *p* < .05) effects of group (a), group‐by‐age interactions (b), and GA (c) on cortical thickness. GA, gestational age; RFT, random field theory

The mean cortical thickness and surface area across the VPT and FT children is shown in Figure 2. Across both groups, there were significant, widespread decreases of cortical thickness with age, spanning the bilateral occipital, frontal and temporal lobes (Figure SS1, Table SS1). Cortical thickness was significantly reduced in VPT compared to FT children in the bilateral midcingulate cortex (Figure 3a, Table 2). Compared to FT children, VPT children had significantly higher cortical thickness in clusters spanning bilateral occipital and orbitofrontal cortices, the bilateral inferior temporal and fusiform gyri, and the right inferior pre‐and post‐central gyri (Figure 3a, Table 2). A significant group‐by‐age interaction was also found in the left fusiform and inferior occipital gyri and right calcarine sulcus, where the cortical thickness of these regions decreased more with age in VPT compared to FT children (Figure 3b, Table 2). Finally, cortical thickness was positively correlated with GA in the VPT children in the right precuneus, cingulate cortex and temporal gyri (Figure 3c, Table 2).

**Table 2:** Clusters with a significant (RFT *p* < .05) effect of group, group‐by‐age interaction, and GA on cortical thickness. AAL regions with at least 100 overlapping vertices with each cluster are reported. For main effects of group, the mean (±*SD*) cortical thickness of the cluster for both the VPT and FT groups are also reported

Contrast	Cluster	*p* Value (RFT‐corrected)	VPT cortical thickness (mm) (mean ± *SD*)	FT cortical thickness (mm) (mean ± *SD*)	AAL regions	# Vertices
FT > VPT	1	2.71e^−3^	3.17 ± 0.17	3.26 ± 0.15	DCG.R	134
	2	0.01	3.09 ± 0.16	3.19 ± 0.15	DCG.L	138
VPT > FT	1	5.57e^−8^	2.99 ± 0.17	2.90 ± 0.13	CAL.R	664
					LING.R	376
					CUN.R	346
					IOG.R	253
					SOG.R	125
	2	5.84e^−8^	2.83 ± 0.16	2.76 ± 0.13	CAL.L	636
					CUN.L	319
					LING.L	171
					MOG.L	149
	3	6.14e‐8	2.84 ± 0.15	2.76 ± 0.12	ORBs.R	728
					REC.R	479
					ORBsm.R	429
					MFG.R	227
					ORBm.R	226
					ORBi.R	164
					SFGm.R	113
	4	7.12e^−8^	3.55 ± 0.21	3.43 ± 0.19	ORBs.L	780
					REC.L	440
					ORBsm.L	359
					SFGm.L	323
					SFG.L	127
					ORBm.L	113
	5	8.87e^−4^	3.75 ± 0.22	3.66 ± 0.20	ITG.R	279
					FFG.R	209
	6	1.85e^−3^	3.43 ± 0.15	3.38 ± 0.14	PoCG.R	249
					ROL.R	209
					PreCG.R	123
	7	1.85e^−3^	3.65 ± 0.20	3.58 ± 0.17	ITG.L	225
					FFG.L	189
Age interaction FT > VPT	1	1.10e^−3^			FFG.L	164
					IOG.L	111
	2	0.02			CAL.R	240
(+) GA	1	1.30e^−4^			PCUN.R	502
					PCG.R	128
	2	5.44e^−4^			MTG.R	552
					ITG.R	135
					STG.R	100
	3	2.05e^−3^			MCG.R	196
					ACG.R	156
	4	0.01			PHG.R	166

Abbreviations: AAL, automated anatomical labeling; FT, full‐term; GA, gestational age; RFT, random field theory; VPT, very preterm.

#### 3.1.3 Surface area

**Figure 4:**
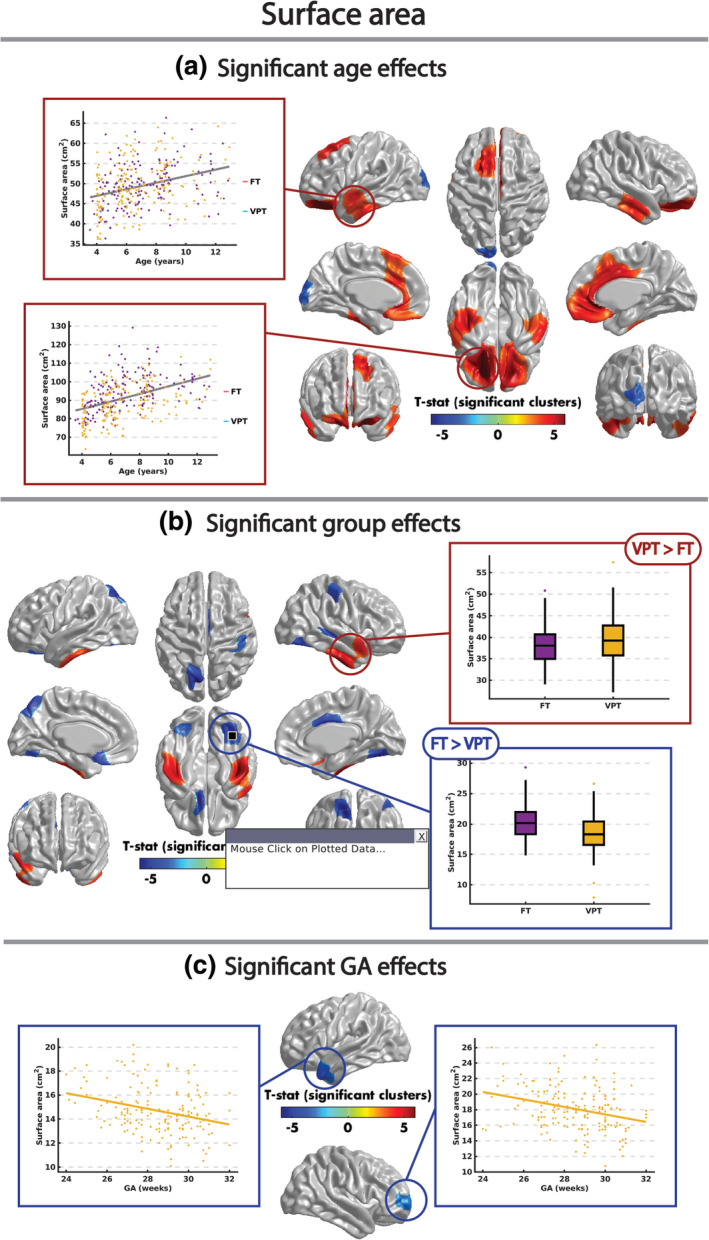
Significant (RFT *p* < .05) effects of age (a), group (b), and GA (c) on surface area. GA, gestational age; RFT, random field theory

Surface area significantly decreased with age in the left calcarine sulcus, cuneus and superior occipital gyrus across both groups (Figure 4a, Table 3). Increases with age were seen in bilateral orbitofrontal and cingulate cortices, middle and inferior temporal gyri, and left fusiform, parahippocampal, and superior frontal and temporal gyri (Figure 4a, Table 3). FT children had significantly larger surface area in the bilateral posterior part of the fusiform gyrus, the left precuneus, superior parietal and orbitofrontal gyri, and gyrus rectus, and the right middle temporal, posterior and middle cingulate gyri (Figure 4b, Table 3). VPT children had significantly larger surface area in the bilateral inferior and middle temporal gyri, superior temporal pole, parahippocampal and anterior fusiform gyri, and the right superior temporal, superior and middle frontal gyri (Figure 4b, Table 3). Surface area in the right superior and middle frontal gyri and left middle temporal gyrus and superior temporal pole correlated negatively with GA in the VPT children (Figure 4c, Table 3). When IQ was included as a covariate in Models 1–4, the reported significant results did not change.

**Table 3:** Clusters with a significant (RFT *p* < .05) effect of age, group, and GA on surface area. AAL regions with at least 100 overlapping vertices with each cluster are reported. For main effects of group, the mean (±*SD*) surface area of the cluster for both the VPT and FT groups are also reported

Contrast	Cluster	*p* Value (RFT‐corrected)	VPT surface area (cm^2^) (mean ± *SD*)	FT surface area (cm^2^) (mean ± *SD*)	AAL regions	# Vertices
(−) Age	1	8.37e^−3^			CAL.L	267
					CUN.L	221
					SOG.L	164
(+) Age	1	2.19e^−8^			SFG.L	696
					ORBs.L	656
					ORBi.L	509
					SMA.L	504
					REC.L	466
					ACG.L	461
					SFGm.L	369
					MFG.L	165
					ORBm.L	148
					ORBsm.L	134
	2	2.20e^−8^			ACG.R	763
					ORBs.R	686
					DCG.R	672
					REC.R	523
					ORBsm.R	458
					SMA.R	378
					ORBi.R	362
					ORBm.R	230
					SFGm.R	151
	3	6.50e^−6^			MTG.L	624
					ITG.L	523
					STG.L	334
					PHG.L	297
					FFG.L	192
	4	7.32e^−4^			MTG.R	678
					ITG.R	509
FT > VPT	1	2.7e^−3^	24.83 ± 3.00	27.59 ± 3.24	PCUN.L	408
					SPG.L	386
					CUN.L	103
	2	3.93e^−3^	18.47 ± 2.77	20.45 ± 2.62	FFG.R	423
					IOG.R	190
	3	0.02	10.64 ± 1.59	11.93 ± 1.55	MTG.R	396
	4	0.03	13.63 ± 1.95	15.15 ± 2.27	PoCG.R	429
					IPL.R	102
	5	0.03	7.98 ± 1.17	8.81 ± 1.12	FFG.L	283
	6	0.04	9.84 ± 1.09	10.78 ± 1.21	REC.L	236
					ORBs.L	201
	7	0.04	6.22 ± 0.86	6.84 ± 0.79	DCG.R	235
VPT > FT	1	2.17e^−4^	39.13 ± 4.91	38.09 ± 4.28	ITG.R	517
					TPOs.R	309
					STG.R	257
					MTG.R	223
					PHG.R	143
					FFG.R	119
	2	3.43e^−3^	22.97 ± 3.18	22.41 ± 2.49	ITG.L	433
					PHG.L	215
					FFG.L	124
(−) GA	1	0.02			TPOs.L	224
					MTG.L	221
	2	0.02			SFG.R	204
					MFG.R	190

Abbreviations: AAL, automated anatomical labeling; FT, full‐term; GA, gestational age; RFT, random field theory; VPT, very preterm.

## 4 DISCUSSION

This is the largest‐to‐date analysis of brain structure in VPT born children compared to full‐term controls, across childhood. The aim was to resolve the discrepancies in the literature and clarify any atypical maturational changes in brain structure related to VPT birth; to do this we included a large cohort of matched VPT and FT children, from early to mid‐childhood and completed analyses on cortical thickness, surface area and deep gray matter volumes. Consistent with prior reports (e.g., Lax et al., 2013; Monson et al., 2016; Zhang et al., 2015), we found reduced TBV in the VPT compared to the full‐term controls across the age range studied (4–12 years), with parallel increases with age in both groups. This demonstrates decreased TBV is associated with VPT birth, but there is an otherwise typical maturational trajectory in TBV from 4 years of age. There were comparable overall gray matter decreases with age, but an age by group interaction in the white matter increases with age, such that similar volumes were seen in the two groups of children by the beginning of adolescence. Continued study of the longitudinal cohorts within this sample would provide crucial evidence as to whether there is catch up in the VPT group in other measures.

Most interesting were the effects in finer measures of brain structure. With cortical thickness and mean surface area, the age‐related changes looked globally similar in both groups (Figure 2), but significant region‐specific group differences emerged. For the cortical thickness, the VPT group showed thicker cortex in frontal, occipital and fusiform gyri, and inferior pre‐ and post‐central regions. As apparent cortical thickness decreases with age start early in childhood (Ducharme et al., 2016; Mills et al., 2016; Raznahan et al., 2011; Remer et al., 2017; Walhovd, Fjell, Giedd, Dale, & Brown, 2017), this suggests that this process is delayed in the VPT group in these brain areas. This is reinforced by the group‐by‐age interactions (Figure 3b) that showed more rapid decreases in occipital regions in the VPT children, and by the report that 3 to 4‐year‐old VPT children to also have widespread increases in cortical thickness (Phillips et al., 2011). The areas with thicker cortex in the VPT were those involved in primary visual processing and detailed “what” visual processing, including faces and letters, of the fusiform (Allison, Mccarthy, Nobre, Puce, & Belger, 1994; McCarthy, Puce, Belger, & Allison, 1999). Slower maturation of these regions could affect the VPT child's processing of complex visual stimuli, impacting both learning and social behaviour. The right hemisphere regions related to vocalization (inferior pre‐ and post‐central gyri) also showed atypical cortical thickness in the VPT group. These areas have been linked to natural speech production, particularly synchronous speech (Alexandrou, Saarinen, Kujala, & Salmelin, 2016; Jasmin et al., 2016). VPT children have been highlighted to have language difficulties (Peña, Pittaluga, & Mehler, 2010; Vohr, 2014), which extend to social communication difficulties (Lowe et al., 2019); thus, slower maturation of this brain area may underlie those commonly reported weaknesses in VPT cohorts.

The underlying biological mechanisms for apparent cortical thinning in childhood are complex and multifaceted (Fjell et al., 2015) and likely include synaptic pruning and intracortical myelination. Both of those processes can be in response to increased utilization of brain regions, as function can sculpt structure (e.g., Draganski et al., 2004; Hyde et al., 2009). Our data suggest that it could be reduced functional use of areas underlying visual and language processing in the VPT that yield the thicker cortex in these areas. However, fully longitudinal designs are requisite to determine cause and effect.

The midcingulate cortex was the only area that showed greater cortical thickness in the FT compared to the VPT children. Decreases in cortical thickness with age of this region are reported (Burgaleta, Johnson, Waber, Colom, & Karama, 2014; Ducharme et al., 2016; Forde et al., 2017), but this part of the cingulate is the thickest and one of the last cortical areas to myelinate (for discussion, see Glasser & Van Essen, 2011). Thus, this may be an area where cortical thinning occurs later, as few studies look at subsections of the cingulate. Further longitudinal data would help clarify this. As has been reported previously, decreasing GA was associated with thinner cortex in a few areas, including the right precuneus, dorsal anterior cingulate and temporal lobe (Lax et al., 2013; Phillips et al., 2011).

In typical development, surface area increases with age until at least 8–9 years and then decreases until early adulthood (Wierenga et al., 2014). Here we report increases across both groups that extended to 12 years of age in frontal, orbital–frontal and temporal areas; these are late maturing brain regions, and thus increases in surface area may continue longer than the average in other brain areas. This is important in terms of highlighting the regional variability in these maturational trends (Burgaleta, Johnson, Waber, Colom, & Karama, 2014; Forde et al., 2017; Wierenga et al., 2014). The only region to show decreasing surface area with age was in the occipital lobe—one of the earliest maturing areas; thus, the decreases were already the dominant trend in both groups of children in this area.

For the significant group differences in surface area, we would argue they are due to the VPT lagging behind the FT on this cubic‐shaped maturation curve. The temporal regions, particularly the inferior temporal, had greater surface area in the VPT group; indicating that VPT children are still on the ascending or top of the maturation curve of surface area, while in the FT surface area was decreasing. In contrast, we found scattered areas where the FT had greater surface area, which suggests that the VPT were lagging on the ascending slope. This model also accounts for the GA effects found in the left temporal pole and frontal lobe; those with the lowest GA were less mature such that the surface area was still at an earlier maturational stage in the curve, and thus still larger than those born closer to 32 weeks GA.

Unlike studies with smaller samples but within the same age range (Grunewaldt et al., 2014; Kesler et al., 2008; Lax et al., 2013; Peterson et al., 2000; Sølsnes et al., 2015), we did not find smaller subcortical volumes in the VPT group. An exception was the striatal percentage volume that was larger in VPT, likely due to the age‐related decreases, and the VPT being less mature. These findings suggest that some of the reported effects in the literature may be due to the age when the subcortical gray matter was compared between VPT and FT participants in the other studies. Alternatively, however, earlier studies (e.g., Kesler et al., 2008; Peterson et al., 2000) did not always correct for TBV, such that decreased deep gray matter volumes would be confounded with the smaller intracranial volumes in the VPT children. There are a number of studies that also report decreased subcortical gray matter in adolescence and adulthood even with correcting for intracranial volume (Aanes, Bjuland, Skranes, & Løhaugen, 2015; Meng et al., 2016), although when Nagy et al. (2009) controlled for TBV, all subcortical differences disappeared. There may, however, be further age‐related changes in these structures and differences may emerge with an older cohort.

Thus, we found significant differences in cortical and subcortical gray matter in VPT compared to FT children, across childhood, which were consistent with a slower maturational process in the VPT group. As the cortical thickness differences were concordant with slower cortical thinning, this may well be an aspect of brain structure where there is some catch up to their FT peers. Similarly, our data showed catch‐up in terms of white matter volumes by 12 years of age, despite significant differences in the children around 4–6 years of age. This model of catch‐up is supported by recent work in adults suggesting that there is accelerated maturation toward adulthood in the VPT (Karolis et al., 2017; Mürner‐Lavanchy et al., 2014). Over childhood, however, the cortical areas that appear to be lagging in development are some that underlie cognitive functions with which the VPT often experience difficulties, including visual and social–emotional processing, and language.

Cortical surface area is believed to be related to the number of mini‐columns, based on the number of progenitor cells in the ventricular zone during embryogenesis (Rakic, 2009), while cortical thickness is driven by the number of neurons, glia and arborization within the columns and pruning (Huttenlocher, 1990). Neuronal migration is largely complete by the time of VPT birth (Raybaud, Ahmad, Rastegar, Shroff, & Al Nassar, 2013), and thus the underpinnings of cortical surface area are already established. In contrast, the cortical growth that contributes to cortical thickness is malleable throughout life. Thus, although there were significant differences in surface area between the groups, as also reported by others (e.g., Nosarti et al., 2008; Zhang et al., 2015), this may explain why, in contrast to cortical thickness, there were no age by group interactions. Cortical thickness, although showing general trends with development, is variable and is not reliably associated with age (Lewis et al., 2018).

Potential limitations to this study include the use of an adult template for cortical surface generation. To date, the CIVET pipeline does not include a child template, and thus the ICBM152 nonlinear template was used. However, as children, particularly before 6 years of age, can exhibit different brain morphology compared to adults (Phan, Smeets, Talcott, & Vandermosten, 2018), future studies are necessary to confirm our results using an age‐specific template. Additionally, the cohort in the present study has incomplete longitudinal data, which prevents construction of within‐subject developmental trajectories; more complete longitudinal data would increase power in the analysis of group‐level trajectories.

## 5 CONCLUSIONS

In summary, we investigated whole‐brain volumes, subcortical grey matter volumes, and cortical thickness and surface area in a large cohort of VPT children spanning early to late childhood, and compared these measures with their FT peers. We found expected decreases in TBV in the VPT compared to FT children, and indications of the VPT children catching up to the FT children in whole‐brain white matter volume. Significant differences in cortical thickness and surface area point to a delayed maturational process in those born VPT, with differences occurring in regions underlying cognitive processes which are known to be affected by preterm birth. Further research would be important to extend these maturational trajectories from childhood into adolescence.

## Supporting information


**Figure S1** Significant (RFT *p* < .05) main effects of age on cortical thickness.
**Table S1:** Clusters with a significant (RFT *p* < .05) main effect of age on cortical thickness. AAL regions with at least 100 overlapping vertices with each cluster are reported.

## Data Availability

The data that support the findings of this study are available from the corresponding author upon reasonable request.
