# Supplementary material for: Mapping the neuroanatomical impact of very preterm birth across childhood
Source: Hum Brain Mapp. 2019 Nov 5;41(4):892–905. doi: 10.1002/hbm.24847 (PMC7267987; doi:10.1002/hbm.24847)
Supplement: Supplementary file 1 — Figure S1 Significant (RFT p < .05) main effects of age on cortical thickness. Table S1: Clusters with a significant (RFT p < .05) main effect of age on cortical thickness. AAL regions with at least 100 overlapping vertices with each cluster are reported. [file HBM-41-892-s001.docx]

**Mapping the neuroanatomical impact of very preterm birth across childhood**

**Supplemental information**

Marlee M. Vandewouw, MASc^1,2^, Julia M. Young, PhD^1,2,3^, Sarah I. Mossad, MA^1,2,3^, Julie Sato, MA^1,2,3^, Hilary A.E. Whyte, MD^4,5^, Manohar M. Shroff, MD^1,5,6^, Margot J. Taylor, PhD^1,2,3,5,6^

^1^Department of Diagnostic Imaging, Hospital for Sick Children, Toronto, Ontario, Canada M5G 1X8

^2^Program in Neurosciences & Mental Health, Hospital for Sick Children, Toronto, Ontario, Canada M5G 1X8

^3^Department of and Psychology, University of Toronto, Toronto, Ontario, Canada M5S 1A1

^4^Department of Neonatology, Hospital for Sick Children, Toronto, Ontario, Canada M5G 1X8

^5^Department of Paediatrics, University of Toronto, Toronto, Ontario, Canada M5S 1A1

^6^Department of and Medical Imaging, University of Toronto, Toronto, Canada M5S 1A1


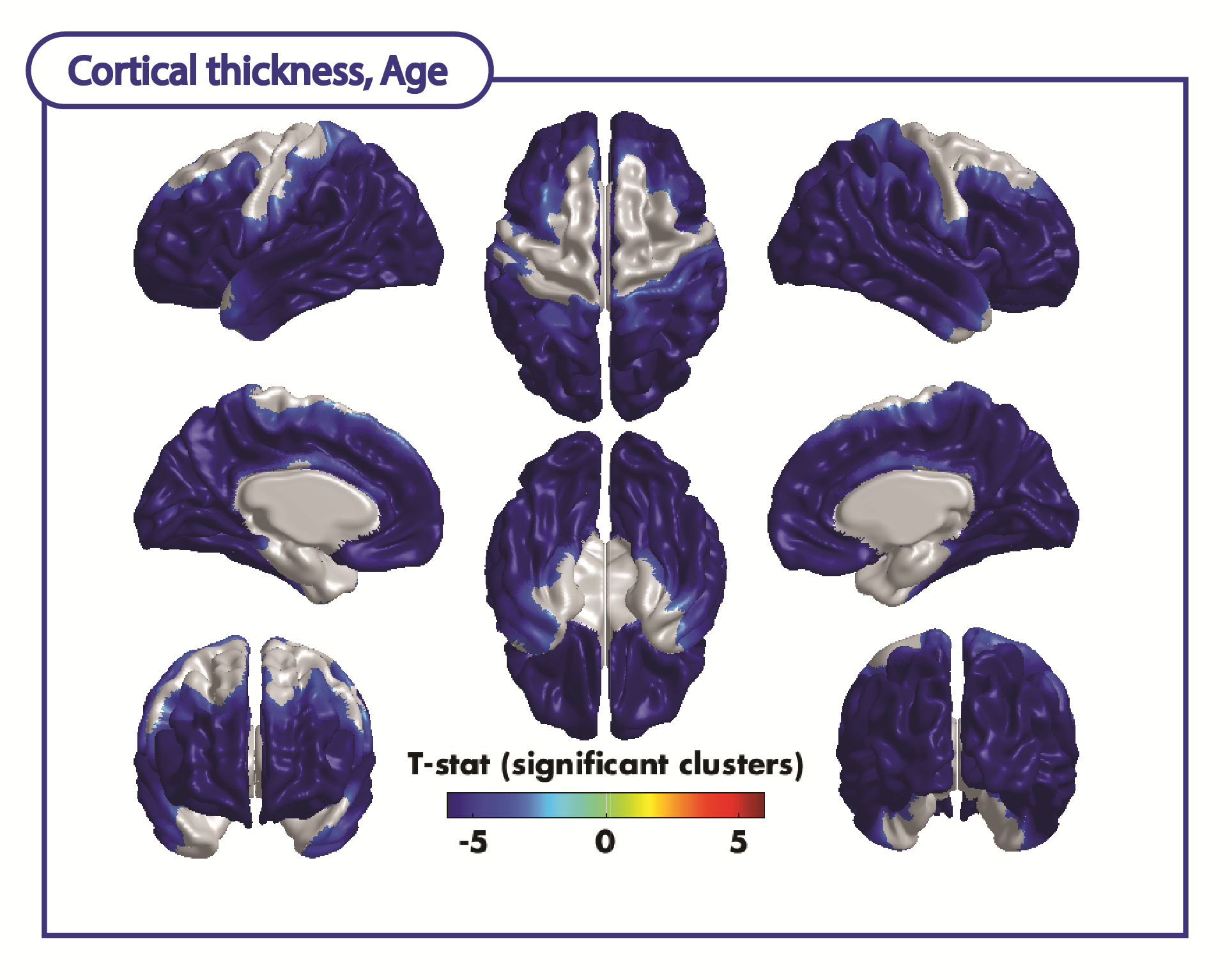


Supplemental Fig. 1: Significant (RFT p < 0.05) main effects of age on cortical thickness.

Supplemental Table 1: Clusters with a significant (RFT p < 0.05) main effect of age on cortical thickness. AAL regions with at least 100 overlapping vertices with each cluster are reported.

| Contrast | Cluster | p-value  (RFT-corrected) | AAL regions | # Vertices |
| --- | --- | --- | --- | --- |
| (-) Age | 1 | 5.10e^-8^ | MTG.L | 2240 |
|  |  |  | MFG.L | 2038 |
|  |  |  | PCUN.L | 1831 |
|  |  |  | MOG.L | 1785 |
|  |  |  | STG.L | 1601 |
|  |  |  | PoCG.L | 1332 |
|  |  |  | CUN.L | 1290 |
|  |  |  | CAL.L | 1199 |
|  |  |  | SPG.L | 1155 |
|  |  |  | SFGm.L | 1114 |
|  |  |  | SFGd.L | 1022 |
|  |  |  | ITG.L | 953 |
|  |  |  | INS.L | 922 |
|  |  |  | ORBs.L | 853 |
|  |  |  | ORBi.L | 828 |
|  |  |  | FFG.L | 817 |
|  |  |  | DCG.L | 802 |
|  |  |  | ANG.L | 768 |
|  |  |  | IFGt.L | 748 |
|  |  |  | SMA.L | 701 |
|  |  |  | IPL.L | 698 |
|  |  |  | LING.L | 697 |
|  |  |  | SMG.L | 689 |
|  |  |  | SOG.L | 634 |
|  |  |  | REC.L | 555 |
|  |  |  | ROL.L | 549 |
|  |  |  | ACG.L | 544 |
|  |  |  | IFGo.L | 542 |
|  |  |  | PHG.L | 528 |
|  |  |  | PCL.L | 526 |
|  |  |  | IOG.L | 489 |
|  |  |  | ORBsm.L | 463 |
|  |  |  | PreCG.L | 461 |
|  |  |  | ORBm.L | 407 |
|  |  |  | HES.L | 312 |
|  |  |  | PCG.L | 230 |
|  |  |  | TPOm.L | 141 |
|  |  |  | TPOs.L | 129 |
|  | 2 | 5.10e^-8­­­­^ | PoCG.R | 2078 |
|  |  |  | MTG.R | 2001 |
|  |  |  | MFG.R | 1986 |
|  |  |  | STG.R | 1873 |
|  |  |  | PCUN.R | 1795 |
|  |  |  | MOG.R | 1360 |
|  |  |  | CUN.R | 1313 |
|  |  |  | SPG.R | 1238 |
|  |  |  | CAL.R | 1172 |
|  |  |  | ITG.R | 1073 |
|  |  |  | DCG.R | 944 |
|  |  |  | INS.R | 926 |
|  |  |  | SMG.R | 926 |
|  |  |  | ACG.R | 869 |
|  |  |  | ORBi.R | 844 |
|  |  |  | FFG.R | 813 |
|  |  |  | SMA.R | 789 |
|  |  |  | ORBs.R | 788 |
|  |  |  | IFGt.R | 782 |
|  |  |  | SFGm.R | 762 |
|  |  |  | ANG.R | 748 |
|  |  |  | SFGd.R | 707 |
|  |  |  | LING.R | 687 |
|  |  |  | IOG.R | 647 |
|  |  |  | SOG.R | 610 |
|  |  |  | ROL.R | 558 |
|  |  |  | PHG.R | 558 |
|  |  |  | PCL.R | 545 |
|  |  |  | IFGo.R | 538 |
|  |  |  | REC.R | 523 |
|  |  |  | PreCG.R | 511 |
|  |  |  | ORBsm.R | 477 |
|  |  |  | ORBm.R | 471 |
|  |  |  | IPL.R | 462 |
|  |  |  | HES.R | 290 |
|  |  |  | PCG.R | 228 |
|  |  |  | TPOs.R | 200 |
